# Integrating Environmental Drivers and Trophic Interactions to Predict Spatial Distribution of High-Risk Marine Organisms at Nuclear Power Plant Cooling Water Intake

**DOI:** 10.3390/ani16081275

**Published:** 2026-04-21

**Authors:** Yunlei Zhang, Xinyue Hu, Linquan Cao, Guize Liu, Changchun Song, Yuan Jin

**Affiliations:** 1 State Environmental Protection Key Laboratory of Coastal Ecosystem, National Marine Environmental Monitoring Center, Dalian 116023, China; ylzhang@nmemc.org.cn (Y.Z.); lqcao0225@163.com (L.C.); laogui117117@163.com (G.L.); 2 College of Construction Engineering, Dalian University of Technology, Dalian 116081, China; 17756323217@163.com (X.H.); songcc@dlut.edu.cn (C.S.)

**Keywords:** joint species distribution model, trophic interactions, cooling water intake, high-risk marine species, coastal ecosystems

## Abstract

Marine organisms that episodically aggregate near coastal nuclear power plant water intakes pose a substantial risk to cooling water security. Predicting the spatial distribution of such high-risk species remains challenging because their occurrence is shaped not only by environmental conditions but also by complex trophic interactions. In this study, we model the habitat distribution of three high-risk nektonic species, Dotted gizzard shad (*Konosirus punctatus*), Japanese swimming crab (*Charybdis japonica*) and squid (*Loligo* sp.), in the cooling water intake area of a coastal nuclear power plant in eastern Liaodong Bay using generalized linear models (GLMs) and joint species distribution models (JSDMs). Based on summer surveys conducted in 2024–2025, we explicitly incorporated trophic linkages among target species, their prey, and predators within JSDMs. Model performance was evaluated using cross-validation based on AUC, RMSE, and coefficient of determination (R^2^). Our results indicate that water depth was the dominant environmental driver for all three species, while chlorophyll-*a* concentration and distance to the intake exerted species-specific effects. By incorporating interspecific trophic associations and environmental responses, JSDMs showed consistently improved predictive performance relative to GLMs, with approximately 1.5-fold higher R^2^ values and 10–30% lower RMSE, while offering enhanced ecological interpretability. The models revealed strong positive associations between target species and both lower-trophic prey and higher-trophic predators, suggesting that top–down and bottom–up processes jointly regulate aggregation dynamics. This study demonstrates that integrating trophic interactions into species distribution modeling substantially improves predictions of high-risk marine species near coastal infrastructure and provides an ecological basis for proactive management of cooling water intake systems.

## Simple Summary

Marine organisms that periodically aggregate near coastal nuclear power plant water intakes can threaten the safe operation of cooling systems. Predicting where these high-risk species occur is difficult because their distributions are influenced not only by environmental conditions but also by interactions within the food web. In this study, we analyzed the spatial distribution of three high-risk nekton species (*Konosirus punctatus*, *Charybdis japonica*, and *Loligo* sp.) in the cooling water intake area of a nuclear power plant in eastern Liaodong Bay. We compared traditional generalized linear models with joint species distribution models that incorporate trophic interactions among species. The results showed that including trophic relationships significantly improved prediction accuracy and helped explain aggregation patterns. Water depth was the most important environmental factor, while prey availability and predator presence also played key roles. Our findings demonstrate that integrating trophic interactions into species distribution models can enhance the prediction of biological risks near coastal infrastructure and support the management of nuclear power plant cooling water intake systems.

## 1. Introduction

Coastal nuclear power plants rely on stable and unobstructed seawater intake systems to ensure the safe operation of cooling circuits. In recent years, however, episodic mass aggregations of marine organisms—such as jellyfish, fish schools, crustaceans, and cephalopods—have increasingly disrupted cooling water intakes worldwide [1,2,3,4]. These biological events are typically characterized by short-term, high-density outbreaks within localized coastal areas, leading to intake blockage, elevated pressure differentials, and, in severe cases, unplanned reactor shutdowns [2,3,5,6,7]. As the frequency and intensity of such biological disturbances continue to increase, there is growing urgency to understand the ecological mechanisms underlying these aggregation events and to develop reliable predictive tools. Although physical measures such as reinforced nets and rotating screens can reduce blockage after organisms enter the intake, they fail to provide early warning of large-scale aggregations. Liaodong Bay, a semi-enclosed basin in the northern Bohai Sea, is particularly vulnerable to such events due to its limited water exchange, intensive industrial development, and the presence of multiple large-capacity cooling water intakes. This combination of physical confinement and anthropogenic pressure creates conditions where episodic mass aggregations can rapidly escalate into operational disruptions, making the bay a critical case for assessing and mitigating biofouling risks.

From an ecological perspective, the spatial distribution of marine organisms is governed by a combination of abiotic environmental filtering and biotic interactions, including predation, competition, and trophic coupling [8,9]. In coastal systems, gradients in water depth, temperature, primary productivity, and distance from shore create heterogeneous habitats that shape species distributions [10,11]. At the same time, trophic interactions across multiple levels of the food web can amplify or suppress local population densities, particularly for mobile nektonic species that respond rapidly to resource availability and predation pressure [12,13]. Consequently, predicting the distribution of high-risk marine species based solely on environmental variables may overlook key biological processes that regulate aggregation dynamics, including trophic interactions (e.g., prey tracking and predator avoidance), social schooling behavior, and interspecific co-occurrence. This oversight leads to reduced predictive accuracy and limited ecological interpretability of traditional models, particularly in highly connected coastal food web systems.

Species distribution models (SDMs) have become an essential tool for quantifying habitat suitability and predicting species occurrence in marine ecosystems [14,15,16]. Traditional SDMs, such as generalized linear models (GLMs), generalized additive models (GAMs), and machine-learning approaches (e.g., Maxent), typically model species independently and assume that species responses to environmental gradients are uncorrelated [17,18]. While these approaches have been widely applied and are computationally efficient, they often fail to account for interspecific dependencies that arise from shared ecological niches or direct biotic interactions. This limitation is particularly relevant in coastal food webs, where closely linked predator–prey relationships and resource coupling can lead to non-independent species distributions [19,20].

Joint species distribution models (JSDMs) provide a conceptual and statistical framework to address this limitation by simultaneously modeling multiple species and their shared responses to environmental variables, while also estimating residual correlations that may reflect biotic interactions or unmeasured environmental factors that are relevant to our coastal study system, including water current velocity, sediment type, and anthropogenic disturbance [21]. By integrating species co-occurrence patterns and environmental drivers, JSDMs offer enhanced explanatory power and improved predictive performance, especially in systems where trophic interactions play a central role. Recent studies have demonstrated the utility of JSDMs in terrestrial and marine ecosystems for community-level inference, biodiversity assessment, and habitat mapping, yet their application to coastal risk management scenarios remains limited [9,22,23].

In the context of cooling water intake safety, most existing studies have focused on engineering countermeasures or descriptive analyses of past biological blockage events [4,24,25]. Ecologically informed predictive models that explicitly link species distributions to food-web structure are still scarce. This knowledge gap constrains the ability of coastal infrastructure managers to anticipate biological risks and to implement proactive mitigation strategies based on ecological early warning signals.

In this study, we address this gap by applying a joint species distribution modeling framework to predict the habitat distribution of three high-risk marine species—Dotted gizzard shad (*Konosirus punctatus*), Japanese swimming crab (*Charybdis japonica*), and squid (*Loligo* sp.)—in the cooling water intake area of a coastal nuclear power plant in eastern Liaodong Bay. These species are characterized by high mobility, mid-to-upper water column occupancy, and a strong propensity to enter intake zones during summer months. Using comprehensive field survey data, we explicitly incorporate trophic linkages between target species, their prey, and their predators into the modeling framework. By comparing the performance of GLMs and JSDMs, we aim to (1) identify the key environmental drivers shaping the distribution of high-risk species, (2) quantify the contribution of trophic interactions to spatial aggregation patterns, and (3) evaluate the added value of JSDMs for ecological prediction in coastal risk-prone environments. Our findings provide ecological insights into the mechanisms underlying biological aggregation near coastal infrastructure and highlight the importance of multi-species approaches for managing human–ecosystem interactions in coastal seas.

## 2. Materials and Methods

### 2.1. Study Area

The study was conducted in the coastal waters surrounding a nuclear power plant located on the eastern coast of Liaodong Bay, northern China. Liaodong Bay is a shallow semi-enclosed shelf system characterized by pronounced gradients in water depth, primary productivity, and coastal hydrodynamics, which together shape complex marine community structures. The cooling water intake area is situated within a nearshore zone that is seasonally influenced by biological aggregation events during summer months [26].

**Figure 1:**
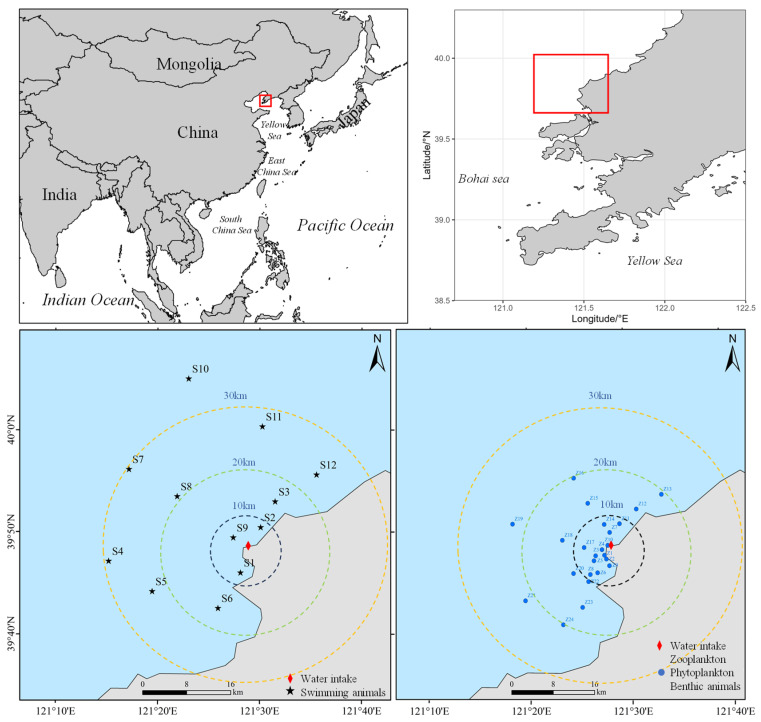
Surveyed sea area surrounding the cooling water intake area of a coastal nuclear power plant located in eastern Liaodong Bay, China. The red boxes represent the study area.

Field surveys were carried out during August 2024 and August 2025, corresponding to the peak period of high-risk biological occurrences. The sampling locations for 2024 and 2025 were the same. A total of 24 stations were established for nekton sampling, 48 stations for plankton and benthic organisms, and 16 stations in the intertidal zone (Figure 1). At each station, biological sampling was conducted concurrently with environmental measurements to ensure temporal consistency between species observations and habitat conditions.

### 2.2. Biological and Environmental Data Collection

#### 2.2.1. Biological Surveys

Plankton samples were collected using shallow-water plankton nets following standardized protocols. Zooplankton were sampled using Type I (entrance diameter 50 cm, mesh size 505 μm) and Type II (entrance diameter 31.6 cm, mesh size: 160 μm) plankton nets, while phytoplankton were collected using a Type III (entrance diameter 37 cm, mesh size 77 μm) net by vertical hauls from the seabed to the surface at a hauling speed of approximately 0.5 m/s. All plankton samples were preserved in 5% buffered formalin and later identified and quantified under an optical microscope. Plankton abundance was expressed as cells per cubic meter (cells/m^3^).

Benthic fauna was quantitatively sampled using a 0.05 m^2^ grab sampler, with a total sampled area of 0.2 m^2^ per station and a penetration depth of 10–20 cm. Sediment samples were sieved through a 0.5 mm mesh, and all retained organisms were preserved in 5% formalin for laboratory identification and enumeration. Benthic organism abundance was expressed as individuals per square meter (ind./m^2^).

Nektonic organisms were surveyed using bottom trawl sampling conducted at each station. Trawling was performed using a single-vessel otter trawl (220 kW) at a speed of 2–3 knots for approximately 1 h per station. The trawl net had an opening width of ~12 m, a height of ~6 m, and a cod-end mesh size of 17 mm. A subsample (~20 kg) of the catch was retained for laboratory analysis; if total catch weight was <20 kg, the entire sample was processed. Species were identified, counted, weighed, and measured. Abundance data (ind./km^2^) for target species were used as response variables in subsequent modeling.

#### 2.2.2. Environmental Variables

**Figure 2:**
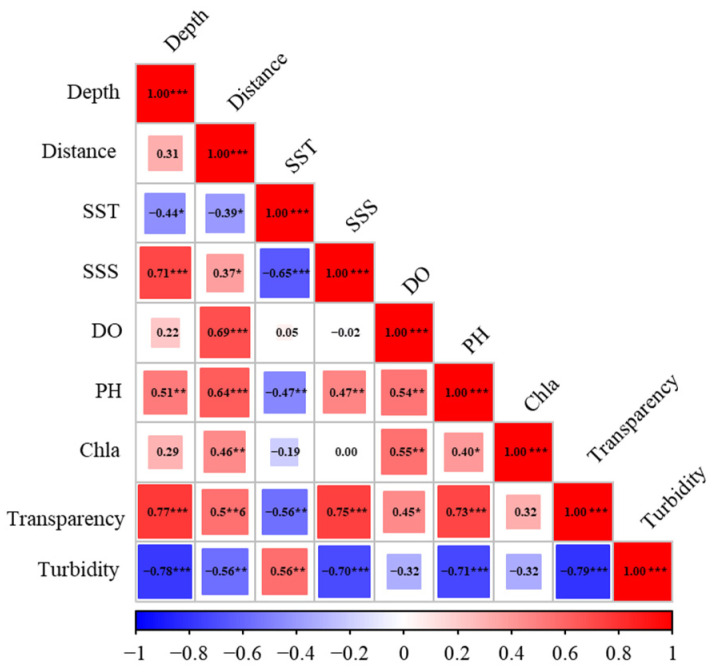
Pearson correlation matrix of environmental variables used for multicollinearity assessment. Distance: linear distance from each sampling station to the cooling water intake; SST, sea surface temperature; SSS, sea surface salinity; DO, dissolved oxygen; Chla, chlorophyll-*a* concentration. *** for *p* < 0.001, ** for *p* < 0.01, * for *p* < 0.05, and no stars for non-significant results (*p* > 0.05).

At each sampling station, environmental parameters were measured in situ using a high-precision multi-parameter CTD profiler (XR-620, RBR Ltd., Ottawa, ON, Canada). Recorded variables included water depth, sea surface temperature (SST), sea surface salinity (SSS), dissolved oxygen (DO), pH, chlorophyll-*a* concentration (Chla), water transparency, and turbidity (Table 1). In addition, the linear distance from each sampling station to the cooling water intake was calculated and included as a spatial explanatory variable to capture potential intake-related gradients. These gradients include changes in hydrodynamics, water quality, and disturbance intensity associated with the operation of the cooling water intake, which may affect the spatial distribution of nektonic species. Based on ecological relevance and data availability, an initial set of nine environmental variables was considered for modeling. Pearson’s correlation analysis was used to reduce multicollinearity among environmental predictors, retaining only variables with |r| < 0.7 [27], which yielded four final predictors: water depth, SST, Chl-a, and distance to the intake (Figure 2).

### 2.3. Target Species and Trophic Structure

**Figure 3:**
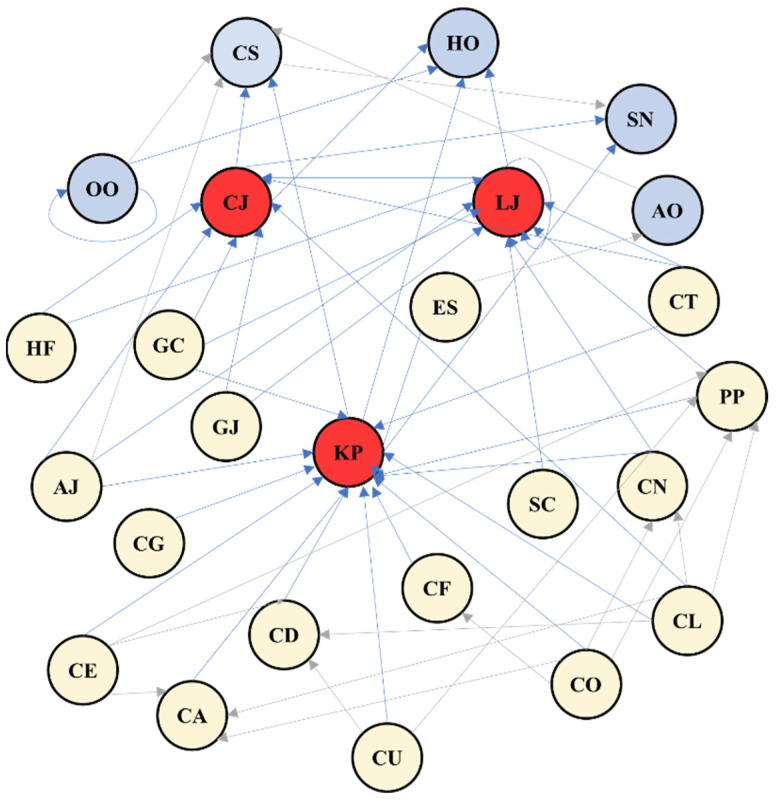
Trophic relationships among species illustrated by directional arrows. Arrows originate from prey and point toward predators. Target species are shown in red, predators in blue, and prey species in yellow.

Three nektonic species were selected as focal high-risk organisms: *Konosirus punctatus*, *Charybdis japonica*, and *Loligo* sp. These species exhibit high mobility, mid-to-upper water column occupancy, and a strong tendency to enter cooling water intake areas during summer, making them particularly relevant for ecological risk prediction [28]. To explicitly incorporate trophic interactions, a simplified food-web structure was constructed based on regional dietary studies and field observations [29,30,31,32] (Figure 3). The trophic network included the three target species, five predator species, and seventeen prey species spanning phytoplankton, zooplankton, benthic invertebrates, and small nekton (Table 2). This trophic information was used to guide species inclusion in the joint modeling framework and to interpret interspecific correlation patterns estimated by the models. To ensure spatial consistency across datasets, abundance data of nektonic organisms were interpolated using kriging so that their spatial locations matched those of other biological groups and environmental variables.

### 2.4. Model Construction

#### 2.4.1. Generalized Linear Models (GLMs)

As a baseline approach, generalized linear models (GLMs) were fitted independently for each target species. Species abundance was used as the response variable, and selected environmental variables were included as predictors. A logit link function with a normal error distribution was applied to relate expected abundance to linear combinations of predictors [33,34]:

(1) $$Y=\alpha +\sum_{i=1}^{n}\beta_{i}x_{i}$$

where *Y* denotes species abundance, *x_i_* represents environmental predictors, *β_i_* are regression coefficients, *α* is the intercept, and *n* is the number of explanatory variables. GLMs were used primarily to assess species–environment relationships and to provide a benchmark for evaluating the performance of joint models.

#### 2.4.2. Joint Species Distribution Models (JSDMs)

To account for interspecific dependencies, joint species distribution models (JSDMs) were constructed using the Generalized Joint Attribute Model (GJAM) framework. GJAM extends GLMs by jointly modeling multiple species responses to environmental predictors while estimating a residual covariance matrix that captures unexplained correlations among species [21,35]. These residual correlations may arise from trophic interactions, shared environmental preferences, or unmeasured ecological processes [19]. In the JSDM framework, species abundance data for all modeled species were combined into a multivariate response matrix, allowing simultaneous estimation of species-specific environmental responses and interspecific associations [19]. Environmental predictors entered the model as fixed effects, while species correlations were represented as random effects through an unstructured covariance matrix. Posterior distributions of model parameters were estimated using Gibbs sampling with 10,000 iterations, discarding the first 1000 iterations as burn-in. The model is available as R package ‘gjam’ version 4.4.2 [19].

### 2.5. Model Evaluation and Validation

Model performance was assessed using repeated cross-validation. For each iteration, 70% of the data were randomly selected as a training dataset, and the remaining 30% were used for validation [36]. This procedure was repeated 100 times to ensure robustness. Model predictive accuracy and explanatory power were evaluated using the root mean square error (RMSE), and coefficient of determination (R^2^) [37]. Models with higher R^2^ values and lower RMSE values were considered to have superior performance. Comparisons between GLMs and JSDMs focused on both predictive accuracy and ecological interpretability.

## 3. Results

### 3.1. Environmental Drivers of Target Species Distributions

**Figure 4:**
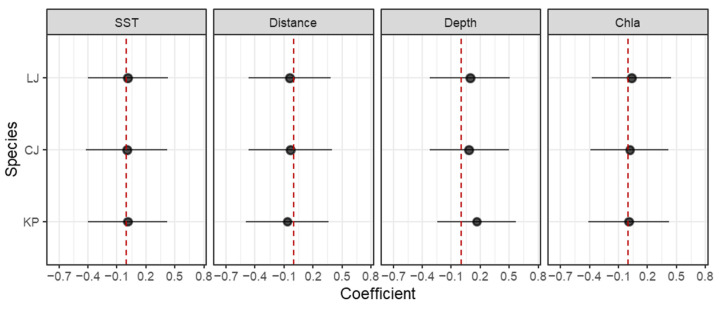
Relative contributions of the four explanatory variables to distributions of three target species in generalized joint attribute model (GJAM). Circles are posterior means and bars are 95% credible intervals (CI). SST: sea surface temperature (°C), Distance: distance to the cooling water intake (km), Depth: water depth (m), Chla: chlorophyll-*a* concentration (mg·m^−3^). KP: *Konosirus punctatus*; CJ: *Charybdis japonica*; LJ: *Loligo* sp. The red dashed lines indicate that the coefficient value is 0.

Across both modeling frameworks, environmental variables exerted consistent but species-specific effects on the spatial distribution of the three high-risk species. In the JSDM results, water depth emerged as the most influential predictor for all target species, exhibiting the highest relative contribution to explained variance (Figure 4). For *Loligo* sp., chlorophyll-*a* concentration and water depth showed strong positive associations with abundance, whereas distance to the intake displayed a negative effect. Sea surface temperature exerted only a minor influence. In contrast, *Charybdis japonica* exhibited a moderate response to depth, with chlorophyll-*a* and distance to the intake contributing comparably but in opposite directions. *Konosirus punctatus* showed the strongest dependence on spatial variables, particularly depth and distance, while responses to temperature and chlorophyll-*a* were comparatively weak.

**Figure 5:**
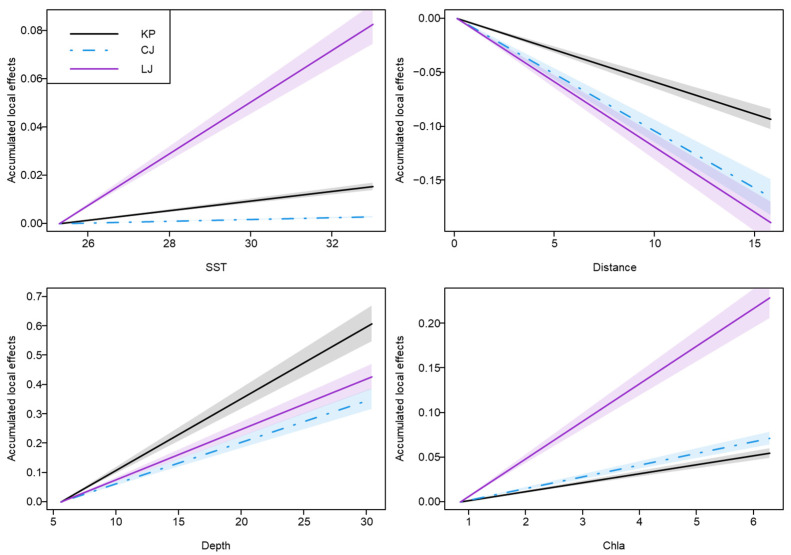
Partial functional relationships for two habitat models of each target species incorporating sea surface temperature (SST, °C), distance to the cooling water intake (Distance, km), water depth (Depth, m) and chlorophyll-*a* concentration (Chla, mg·m^−3^) as explanatory variables. The shading represents the 95% credible intervals. KP: *Konosirus punctatus*; CJ: *Charybdis japonica*; LJ: *Loligo* sp.

The three target species exhibited similar yet species-specific response patterns to the environmental explanatory variables (Figure 5). The results indicated that *Konosirus punctatus* exhibited statistically significant positive responses water depth, and chlorophyll-*a* concentration, together with a significant negative response to distance from the water intake (*p* < 0.05). This reflects its environmental preference for inshore, deeper, and highly productive habitats. For *Loligo* sp., the positive associations with water depth and chlorophyll-*a* concentration, and negative association with distance to the intake were significant (*p* < 0.05), while the response to sea surface temperature was not significant (*p* > 0.05). For *Charybdis japonica*, the positive response to chlorophyll-*a* concentration and negative response to distance to the intake were significant (*p* < 0.05), while its response to water depth and sea surface temperature was moderate and not statistically significant (*p* > 0.05). This pattern might be attributed to its biological traits as a widespread species with strong environmental tolerance, as well as the exclusion of key environmental drivers governing its distribution—such as sediment type—from our model predictors.

### 3.2. Interspecific Associations and Trophic Structure Inferred by JSDM

**Figure 6:**
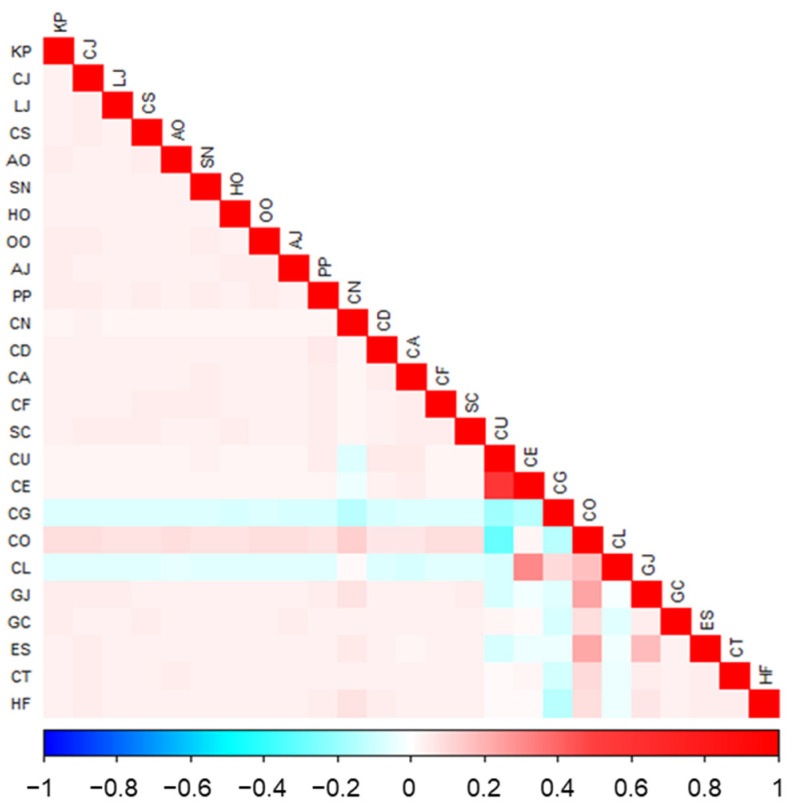
Species trophic interactions identified from generalized joint attribute model (GJAM) after accounting for environmental effects. Each cell represents a pairwise interactions among species. The species names are shown in Table 2.

A key distinction between modeling approaches was the ability of JSDMs to quantify interspecific associations after accounting for environmental effects. Such patterns were not detectable using GLMs, which assume species independence and therefore cannot estimate covariance structures among species. The estimated residual correlation matrix revealed predominantly positive associations among the three target species and most prey and predator species (Figure 6). These associations suggest coordinated responses to shared resources and trophic coupling across multiple levels of the food web. The three target species formed a tightly connected positive correlation cluster with nektonic predators and zooplankton prey, indicating that both bottom–up resource availability and top–down predation pressure contribute to aggregation dynamics. In contrast, a small number of phytoplankton and benthic taxa exhibited weak negative correlations with selected prey species, which may reflect differences in habitat preferences between these taxa, or potential competitive exclusion and niche differentiation in cases where taxa share similar ecological niches.

### 3.3. Model Performance Comparison

**Figure 7:**
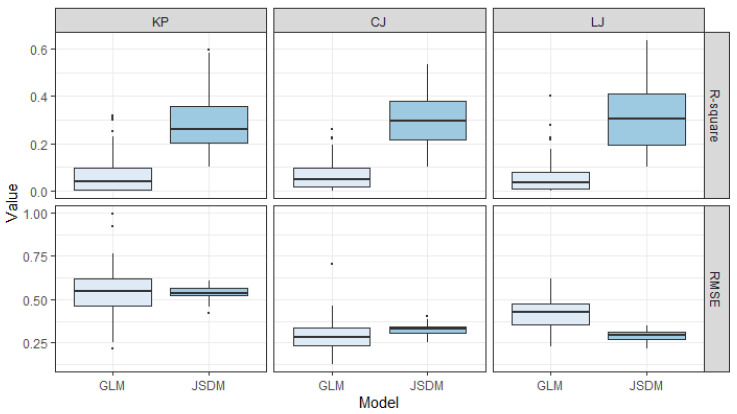
Estimated variance in the abundance of the three target species predicted by the generalized linear model (GLM) and the generalized joint attribute model (GJAM), based on the results of 100 cross-validations. KP: *Konosirus punctatus*; CJ: *Charybdis japonica*; LJ: *Loligo* sp.

Cross-validation results revealed clear differences in predictive performance between GLMs and JSDMs (Figure 7). For all three target species, JSDMs consistently achieved higher coefficients of determination (R^2^) than GLMs. The improvement was most pronounced for *Loligo* sp., for which JSDMs exhibited substantially enhanced predictive performance. For *Konosirus punctatus* and *Charybdis japonica*, the two models yielded comparable RMSE values in spatial distribution predictions; however, JSDMs consistently explained a larger proportion of variance, as indicated by markedly higher R^2^ values. On average, JSDMs increased explanatory power by approximately 1.5-fold relative to GLMs, while reducing RMSE by 10–30%, depending on species.

### 3.4. Spatial Prediction of Species Abundance

**Figure 8:**
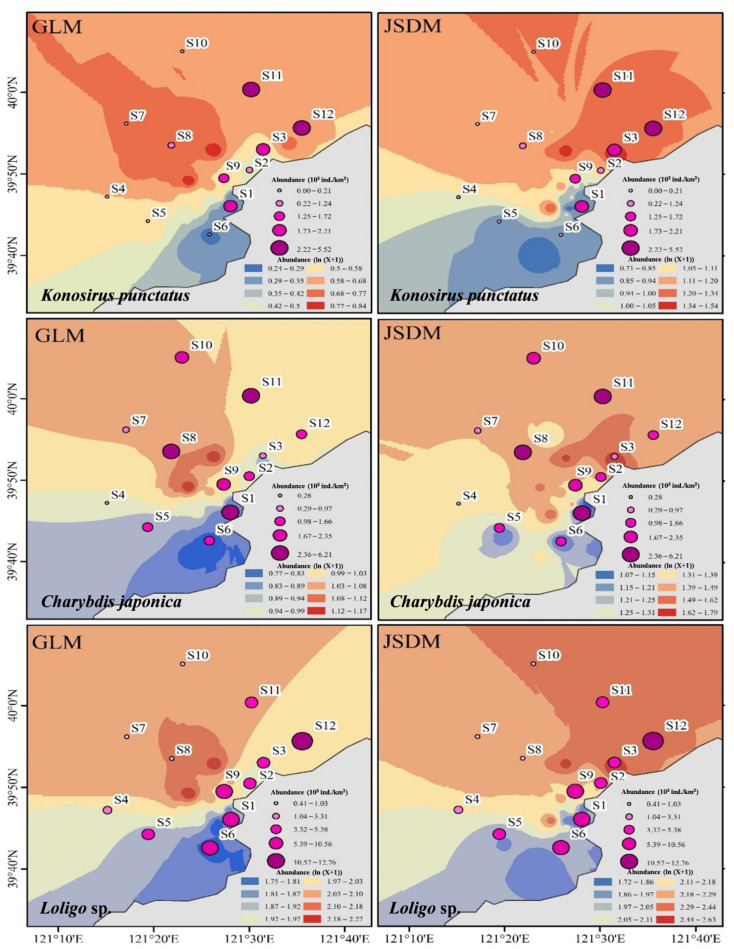
Spatial distributions of three target species near the cooling-water intake of a coastal nuclear power plant in eastern Liaodong Bay, China, as predicted by the generalized linear model (GLM) and the generalized joint attribute model (GJAM). Circles indicate observed abundances at sampling sites (×10^3^ ind.km^−2^). Predicted abundances (ln(X + 1)) are shown as colored contour lines, with spatial interpolation of model outputs performed using ordinary kriging.

Spatial predictions derived from GLMs and JSDMs exhibited broadly similar large-scale patterns but differed substantially in fine-scale structure (Figure 8). GLM-based predictions tended to generate smoother abundance surfaces with elongated high-density zones, occasionally extending into areas unsupported by observations. In contrast, JSDM-based predictions produced more spatially coherent and compact high-density regions that aligned more closely with observed abundance peaks.

For *Konosirus punctatus*, JSDMs more clearly delineated core aggregation areas near the intake, while avoiding excessive smoothing at habitat boundaries. For *Charybdis japonica*, JSDMs reduced false-positive hotspots present in GLM predictions, particularly at stations with low observed abundance. For *Loligo* sp., JSDMs captured a concentrated nearshore–offshore transition zone consistent with its ecological niche, whereas GLMs underestimated abundance at intermediate-depth stations.

## 4. Discussion

### 4.1. Advantages of JSDMs for Nuclear Power Plant Risk Assessment

Predicting species distributions using habitat-based models is critically important for biological risk prevention, early warning, and spatial planning in cooling-water intake areas of nuclear power plants in the eastern Liaodong Bay. In this study, 25 marine species were analyzed, with three high-risk taxa *K. punctatus*, *C. japonica*, and *Loligo* sp.—selected as focal species. By explicitly incorporating trophic information from both prey and predator assemblages, we constructed and compared generalized linear models (GLMs) and joint species distribution models (JSDMs) to evaluate their predictive performance. The results clearly indicate that JSDMs consistently outperform GLMs in predicting the spatial distributions of all three target species, highlighting their suitability for applied ecological risk assessment in nuclear power plant intake zones.

The superior performance of JSDMs stems from their ability to integrate environmental drivers with multispecies co-occurrence patterns and community assembly processes within a unified statistical framework [38,39,40]. Although GLMs and other widely used SDMs—such as Maxent and generalized additive models (GAMs)—have been extensively applied to identify key environmental controls on species distributions [41], they rely on the assumption that species respond independently to environmental gradients. In contrast, JSDMs explicitly model interspecific dependencies through a shared covariance structure, allowing species–environment relationships to be disentangled from residual associations that may arise from trophic interactions, dispersal limitation, or unmeasured environmental gradients [42,43]. While this comes at the cost of increased computational complexity and more demanding model specification, it provides a more realistic representation of ecological processes in complex marine systems.

In coastal ecosystems characterized by strong food-web coupling and pronounced spatial heterogeneity, species distributions are rarely independent [19,44]. The strong positive associations observed among focal species, their prey, and their predators suggest that aggregation events near the cooling-water intake are jointly regulated by bottom–up resource availability and top–down predation pressure. By accounting for these dependencies, JSDMs improve parameter estimation and predictive accuracy for individual species by using shared information across the entire community. Consequently, JSDMs demonstrated higher explanatory power (R^2^) and lower prediction error (RMSE) than GLMs for all three high-risk species. These results underscore the substantial value of joint species distribution modeling for nuclear power plant risk assessment, where accurate prediction of multispecies aggregation dynamics is essential for proactive management and mitigation of biological intake hazards.

### 4.2. Environmental Drivers and Habitat Differentiation of High-Risk Species

Community stability in natural ecosystems is closely linked to the strength of interspecific associations, with stronger associations generally conferring greater stability [9]. This study focused on nektonic organisms, which are simultaneously regulated by top-down predation from higher trophic levels and bottom-up control via planktonic food resources [45,46]. By integrating interactions from both trophic directions, we strengthened the predictive framework for the three focal species. Moreover, by explicitly incorporating the cooling-water intake of the nuclear power plant as a spatial reference, environmental variables such as distance to the intake, water depth, sea surface temperature, and chlorophyll-*a* concentration were included, thereby improving the specificity, accuracy, and practical relevance of the predictions.

Among all environmental predictors, water depth emerged as the most influential variable governing the distributions of all three target species. This finding is consistent with the geomorphological characteristics and ecological processes of the Bohai Sea [47,48]. Depth gradients in shallow continental shelf systems are often associated with thermoclines, haloclines, dissolved oxygen stratification, and spatial heterogeneity in sediment types, all of which shape species-specific habitat preferences [49]. *K. punctatus*, a coastal warm-water fish, preferentially occupies shallow waters [50], whereas *Loligo* sp. exhibits a stronger association with the mid-to-upper water column, with depth exerting an indirect regulatory effect. In contrast, the distribution of *C. japonica* is constrained primarily by suitable benthic substrates [51].

Chlorophyll-*a* concentration exerted a particularly strong positive effect on *Loligo* sp., indicating a high dependence on primary productivity and the food-web base, and suggesting vulnerability to trophic cascades driven by changes in lower trophic levels [31]. In comparison, *K. punctatus* and *C. japonica* were more strongly controlled by physical spatial factors, reflecting greater habitat selectivity rather than active resource tracking. Both species showed pronounced spatial aggregation near the intake area and a tendency to shift toward deeper waters. Meanwhile *Loligo* sp. exhibited no clear preference with respect to intake distance or depth, reflecting a nomadic, resource-tracking generalist strategy in which its distribution is mainly driven by the spatial distribution of zooplankton prey rather than fixed habitat features. These contrasting patterns highlight distinct ecological strategies among the focal species [52], which have important implications for targeted risk management.

### 4.3. Role of Species Interactions and Implications for Management Applications

The integration of interspecific interactions represents the core advantage of JSDMs over traditional single-species models [53]. By constructing a trophic network comprising five predator species and seventeen prey species, our model revealed both positive and negative interaction patterns between the focal species and their adjacent trophic levels. *Loligo* sp. exhibited significant positive correlations with *Sagitta crassa* and *Coscinodiscus oculus-iridi*, reflecting its dual dependence on zooplankton and primary producers. In contrast, negative correlations with *Coscinodiscus granii* and *Chaetoceros lorenzianus*, and conspecifics may indicate competitive exclusion or prey selectivity. Highly positive species associations suggest the presence of strong mutual support mechanisms within the system, contributing to higher biodiversity and ecosystem stability [9,54]. However, excessive reliance on specific environmental conditions may increase community sensitivity to external disturbances. Therefore, integrating both environmental and biological factors yields a more comprehensive and ecologically realistic predictive framework, supporting the rationality of model-based inference in complex marine ecosystems.

By incorporating latent factors, JSDMs capture unobserved niche structures such as common predation pressure, hidden environmental gradients, or dispersal limitations [55]. This capability enhances predictions for rare or low-frequency species and mitigates overfitting in small-sample contexts. Notably, JSDMs avoided the spurious hotspots produced by GLMs at marginal sampling sites, such as those southwest of the intake, thereby improving prediction robustness and ecological plausibility. This is because GLMs are single-species models that assume species independence and therefore cannot leverage co-occurrence information from trophically linked species to constrain predictions. Consequently, at marginal sites with limited environmental data, GLMs tend to over-extrapolate species–environment relationships, yielding false high-abundance predictions. In contrast, GLMs assume independence among species and neglect the non-random structure of community co-occurrence [56], often leading to excessive spatial smoothing and poor delineation of ecological boundaries.

For example, the U-shaped distribution pattern of *Loligo* sp. was oversimplified in the GLM but preserved in the JSDM, reflecting its migratory behavior from offshore to nearshore waters. This finding emphasizes the necessity of accounting for spatial aggregation dynamics and species-specific responses when conducting biological risk assessments for nuclear power plant cooling systems. Although JSDMs entail higher computational complexity and stricter requirements for model specification, their outputs extend beyond abundance prediction to include reconstruction of interaction networks, identification of key drivers, and quantification of uncertainty. These features are critical for developing targeted biological mitigation strategies. If high-density aggregations of risk species are predicted, plant operators may proactively implement measures such as intake net deployment or flow adjustment to reduce blockage risks [57].

Nevertheless, several limitations warrant consideration. The current analysis is based solely on summer survey data and does not capture seasonal migration or interannual variability. Future research should establish long-term monitoring programs and incorporate dynamic JSDMs or state-space models to better represent population dynamics [58]. Another limitation of this study is the kriging interpolation of nekton abundance data to match the sampling stations of plankton and benthic organisms. This interpolation may introduce artificial spatial smoothing errors, which could potentially strengthen or weaken the fine-scale interspecific trophic associations estimated by the JSDM. Additionally, although multicollinearity was addressed using Pearson’s correlation, important drivers such as current velocity, sediment type, and anthropogenic disturbance may have been omitted. Integrating remote sensing products and GIS-based multi-source data fusion will further enhance model performance in future studies.

## 5. Conclusions

This study demonstrates that JSDMs offer clear statistical and ecological advantages over traditional single-species approaches for predicting the distributions of aggregation-prone marine organisms in coastal ecosystems. By integrating shared environmental responses and interspecific dependencies, JSDMs capture key species–environment relationships while accounting for residual associations arising from trophic interactions and unobserved processes. Across the three high-risk nektonic species examined, water depth emerged as the dominant environmental driver, whereas chlorophyll-*a* concentration and spatial proximity showed species-specific effects. Importantly, incorporating interspecific covariance markedly improved predictive accuracy and robustness by reducing spurious spatial hotspots and enhancing model performance under spatial heterogeneity and limited sampling. The inferred positive trophic associations indicate that aggregation dynamics are jointly regulated by bottom–up resource availability and top–down control. Overall, JSDMs provide a flexible and interpretable framework for community-level inference and applied ecological forecasting in complex coastal environments. Future work incorporating temporal dynamics and additional drivers may further enhance their predictive power and ecological realism.

## Figures and Tables

**Table 1:** Environmental explanatory variables and code in modelling analysis.

Variable	Abbreviation	Units	Type	Mean (Min, Max)
sea surface temperature	SST	°C	environmental	27.00 (25.30, 33.00)
sea surface salinity	SSS	PSU	environmental	29.50 (29.20, 29.80)
dissolved oxygen	DO	mg/L	environmental	6.31 (6.00, 7.32)
chlorophyll-*a* concentration	Chla	mg/m^3^	environmental	3.00 (0.82, 6.28)
pH	pH	/	environmental	8.05 (7.92, 8.24)
water transparency	Transparency	m	environmental	2.81 (0.50, 6.00)
turbidity	Turbidity	NTU	environmental	5.06 (0.96, 9.99)
distance to the cooling water intake	Distance	km	spatial	11.64 (0.30, 31.57)
water depth	Depth	m	spatial	16.09 (5.60, 30.40)

**Table 2:** Description, common name, scientific name, and name code of the three target species and their main prey and predators.

Description	Latin Name	Common Name	Name Code
Target species	*Charybdis japonica*	Japanese swimming crab	CJ
	*Konosirus punctatus*	Dotted gizzard shad	KP
	*Loligo* sp.	Squid	LJ
Predator	*Chaeturichthys stigmatias*	Finespot goby	CS
	*Acanthogobius ommaturus*	Javeline goby	AO
	*Scomberomorus niphonius*	Japanese Spanish mackerel	SN
	*Hexagrammos otakii*	Fat greenling	HO
	*Oratosguilla oratoria*	Mantis Shrimp	OO
prey	*Alpheus japonicus*	Japanese snapping shrimp	AJ
	*Paracalanus parvus*	Small marine copepod	PP
	*Calanus sinicus*	Asian calanoid copepod	CN
	*Centropages dorsispinatus*	A species of centropagid copepod	CD
	*Centropages abdominalis*	Centropagid copepod	CA
	*Corycaeus affinis*	Corycaeid copepod	CF
	*Sagitta crassa*	Arrowworm	SC
	*Coscinodiscus subtilis*	Centric diatoms	CU
	*Coscinodiscus asterromphalus*		CE
	*Coscinodiscus granii*		CG
	*Coscinodiscus oculus-iridis*		CO
	*Chaetoceros lorenzianus*	Chain-forming diatom	CL
	*Goniada japonica*	A species of polychaete worm	GJ
	*Glycera chirori*	A species of bloodworm	GC
	*Eogammarus sinensis*	A gammarid amphipod species	ES
	*Cycladicama tsuchii*	Small bivalve mollusk	CT
	*Heteromastus filiforms*	Capitellid worm	HF

## Data Availability

The original contributions presented in the study are included in the Supplementary Material; further inquiries can be directed to the corresponding author.

## Supplementary Materials

The following supporting information can be downloaded at: https://www.mdpi.com/article/10.3390/ani16081275/s1. Figure S1: The trace plot of beta (response to environmental factors) coefficient thinned chains in generalized joint attribute model (GJAM). Figure S2: The trace plot of correlation thinned chains in generalized joint attribute model (GJAM). Figure S3: Ordinate data from GJAM object using correlation corresponding to response matrix E (correlation structure in response to the environment). Results return eigenvalues and eigenvectors using principle component analysis (PCA) in panel (a), and return three non-metric multidimensional scale (NMDS) dimensions using NMDS in panel (b). Figure S4: Joint data prediction for the 25 species. Frequency of observations in abundance (ln(Y + 1)) is shown at the base of graphs (pink bars). Width of boxes adjusts to accommodate equal numbers of observations. Symbols indicate median (horizontal line) 68% (box) and 95% (whisker). Dashed lines are 1:1 (diagonal) and mean of the true values (horizontal). Figure S5: Inverse prediction of (a) sea surface temperature (SST), (b) distance from the water intake point (Distance), (c) water depth (Depth), and (d) Chlorophyll-*a* concentration (Chla). Boxes and whiskers are 68% and 95% predictive intervals; mid lines are means. The distribution of data is shown as histograms. Width of boxes adjusts to accommodate equal numbers of observations. In all panels, the horizontal axis is observed and the vertical axis is predicted. Dashed lines are 1:1 (diagonal) and mean of the true values (horizontal).
