# Supplementary material for: Integrating Environmental Drivers and Trophic Interactions to Predict Spatial Distribution of High-Risk Marine Organisms at Nuclear Power Plant Cooling Water Intake
Source: Animals (Basel). 2026 Apr 21;16(8):1275. doi: 10.3390/ani16081275 (PMC13113618; doi:10.3390/ani16081275)
Supplement: Supplementary file 1 [file animals-16-01275-s001.zip › animals-4233467-supplementary.pdf]

Supplementary materials for

Integrating environmental drivers and trophic interactions to predict spatial distribution of high-risk marine organisms at nuclear power plant cooling water intake

Additional information of the generalized joint attribute model (GJAM) develop was provided, including the convergence diagnostics of the fitted model (Figure S1-S2), and model evaluation (Figure S3-S5).

Generalized Joint Attribute Model develop

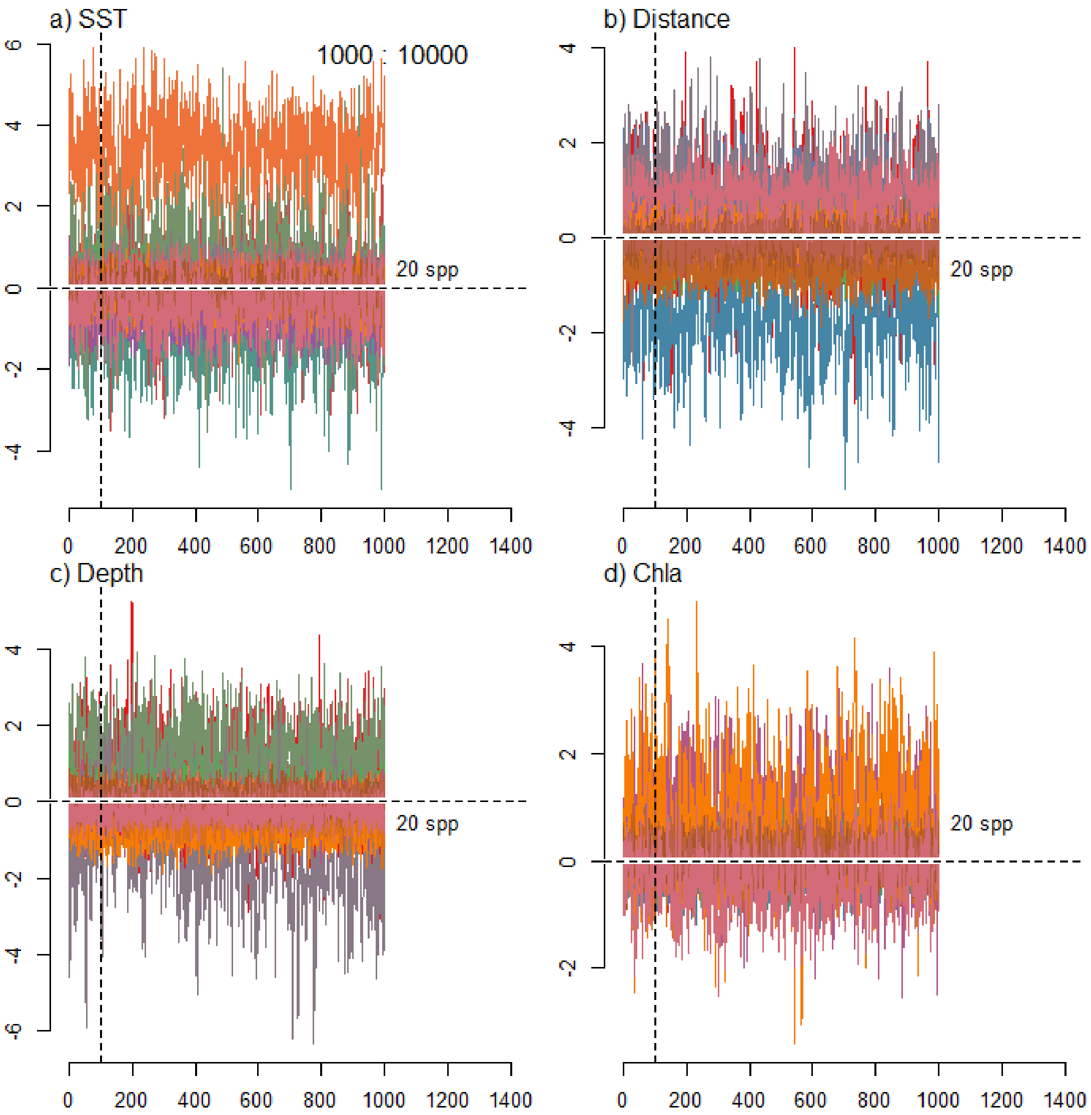

Figure S1. The trace plot of beta (response to environmental factors) coefficient thinned chains in generalized joint attribute model (GJAM).

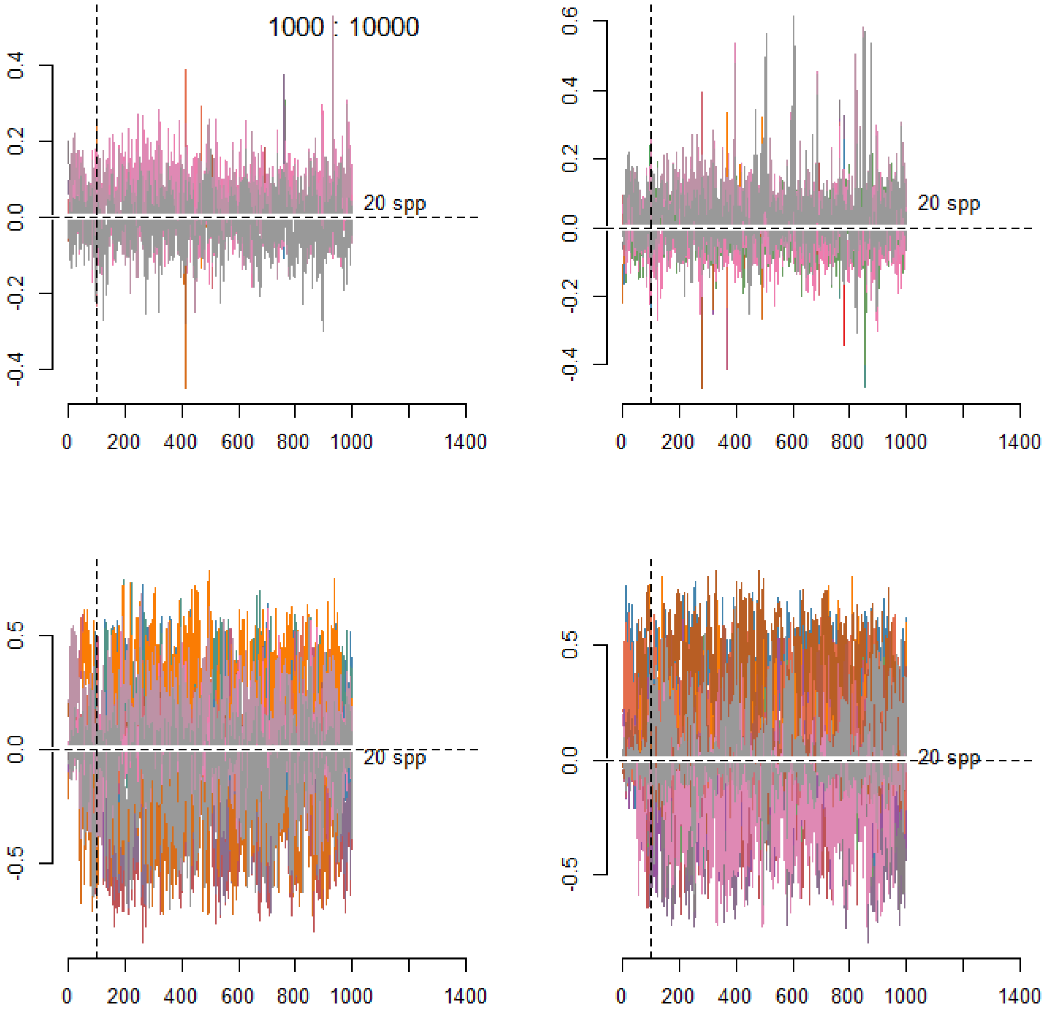

Figure S2. The trace plot of correlation thinned chains in generalized joint attribute model (GJAM).

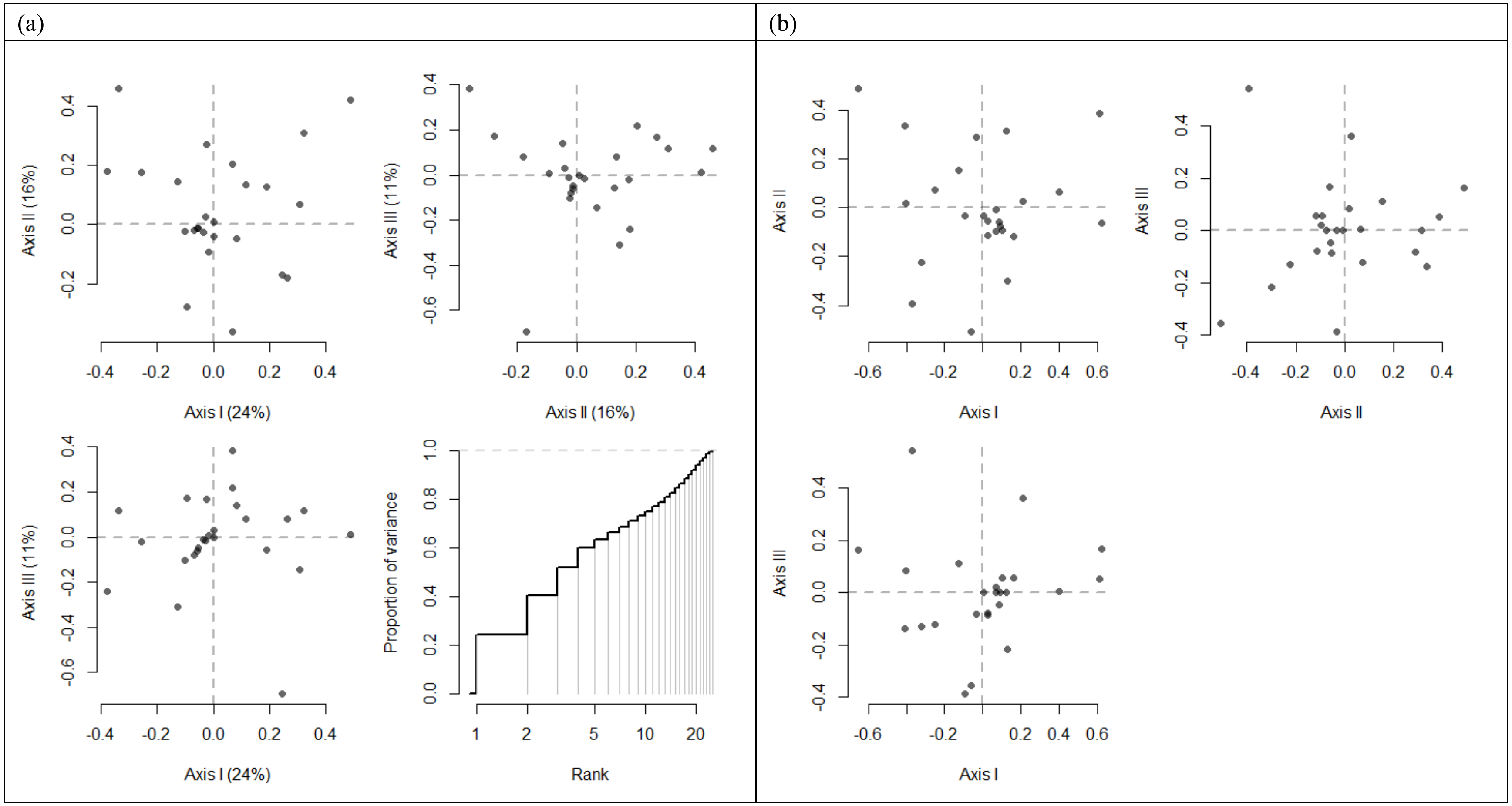

Figure S3. Ordinate data from GJAM object using correlation corresponding to response matrix E (Correlation structure in response to the environment). Results return eigenvalues and eigenvectors using principle components analysis (PCA) in panel (a), and return three non-metric multidimensional scale (NMDS) dimensions using NMDS in panel (b).

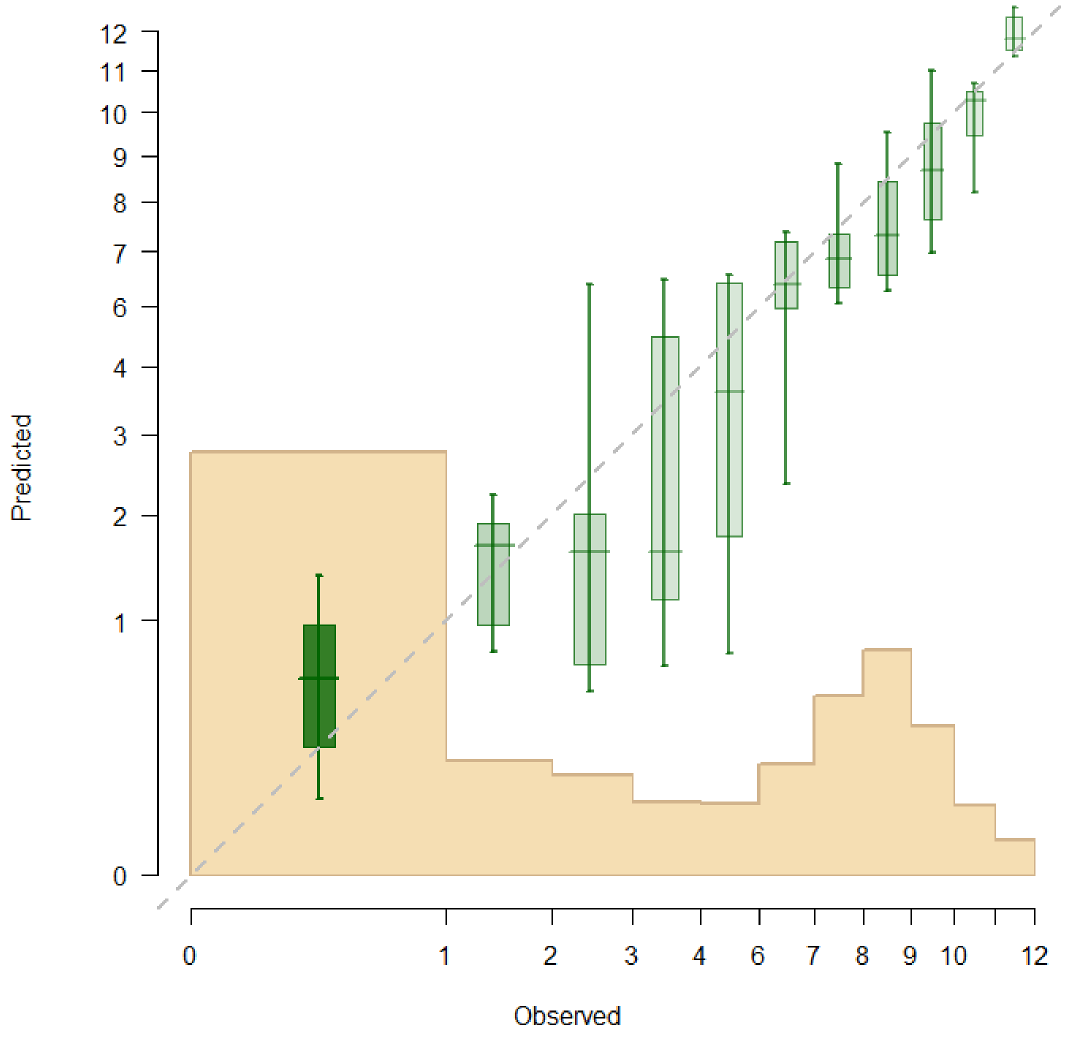

Figure S4. Joint data prediction for the 25 species. Frequency of observations in abundance (ln(Y+1)) is shown at the base of graphs (pink bars). Width of boxes adjusts to accommodate equal numbers of observations. Symbols indicate median (horizontal line) 68% (box) and 95% (whisker). Dashed lines are 1:1 (diagonal) and mean of the true values (horizontal).

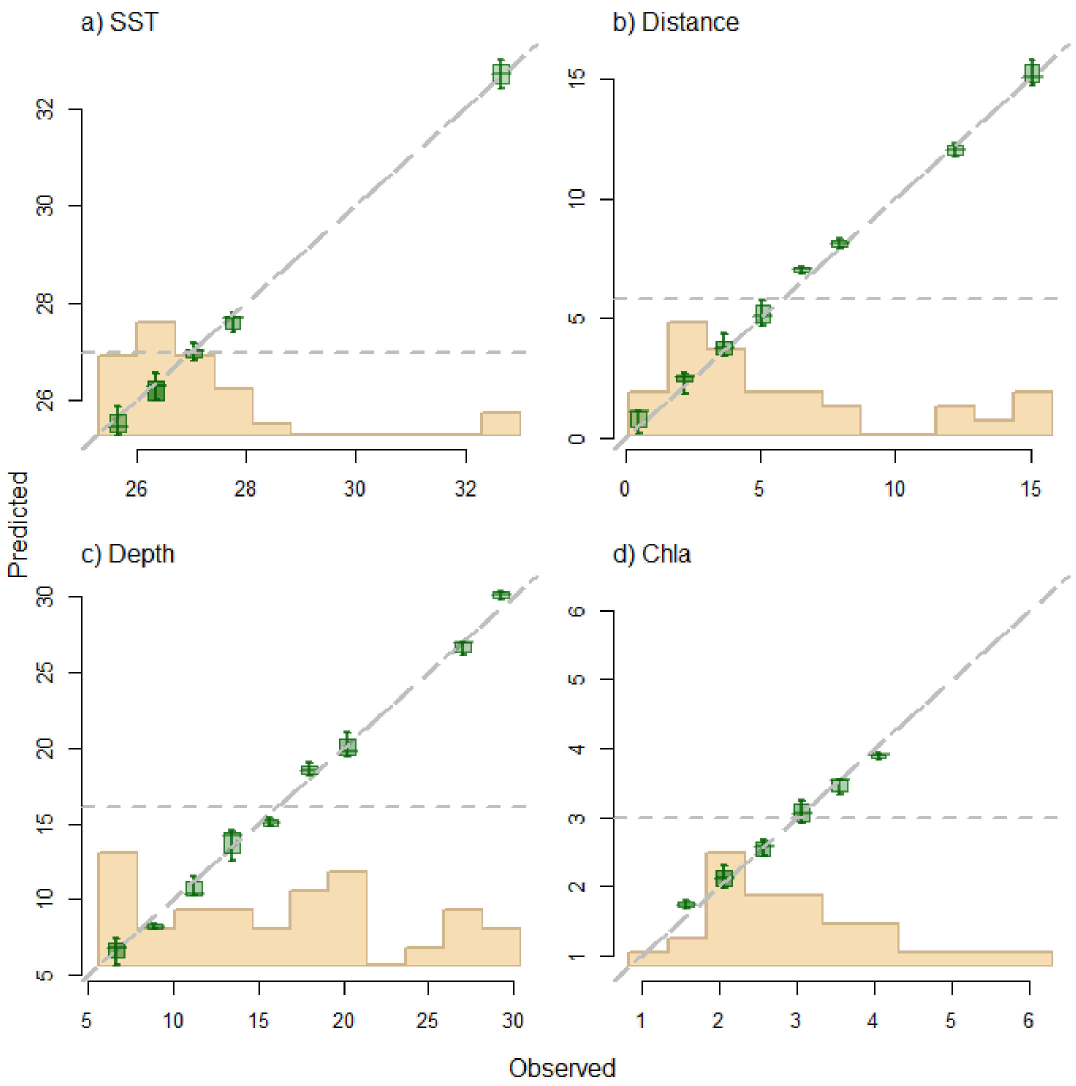

Figure S5. Inverse prediction of (a) sea surface temperature (SST), (b) distance from the water intake point (Distance), (c) water depth (Depth), and (d) Chlorophyll-a concentration (Chla). Boxes and whiskers are 68% and 95% predictive intervals, mid lines are means. The distribution of data is shown as histograms. Width of boxes adjusts to accommodate equal numbers of observations. In all panels, the horizontal axis is observed and the vertical axis is predicted. Dashed lines are 1:1 (diagonal) and mean of the true values (horizontal).
